# From rest to repair: Safeguarding genomic integrity in quiescent cells

**DOI:** 10.1016/j.dnarep.2024.103752

**Published:** 2024-08-20

**Authors:** Chin Wei Brian Leung, Jacob Wall, Fumiko Esashi

**Affiliations:** Sir William Dunn School of Pathology, South Parks Road, Oxford, UK

**Keywords:** DNA repair, Quiescence, Genome stability, Transcription, Metabolism

## Abstract

Quiescence is an important non-pathological state in which cells pause cell cycle progression temporarily, sometimes for decades, until they receive appropriate proliferative stimuli. Quiescent cells make up a significant proportion of the body, and maintaining genomic integrity during quiescence is crucial for tissue structure and function. While cells in quiescence are spared from DNA damage associated with DNA replication or mitosis, they are still exposed to various sources of endogenous DNA damage, including those induced by normal transcription and metabolism. As such, it is vital that cells retain their capacity to effectively repair lesions that may occur and return to the cell cycle without losing their cellular properties. Notably, while DNA repair pathways are often found to be downregulated in quiescent cells, emerging evidence suggests the presence of active or differentially regulated repair mechanisms. This review aims to provide a current understanding of DNA repair processes during quiescence in mammalian systems and sheds light on the potential pathological consequences of inefficient or inaccurate repair in quiescent cells.

## 1 Introduction

**Fig. 1:**
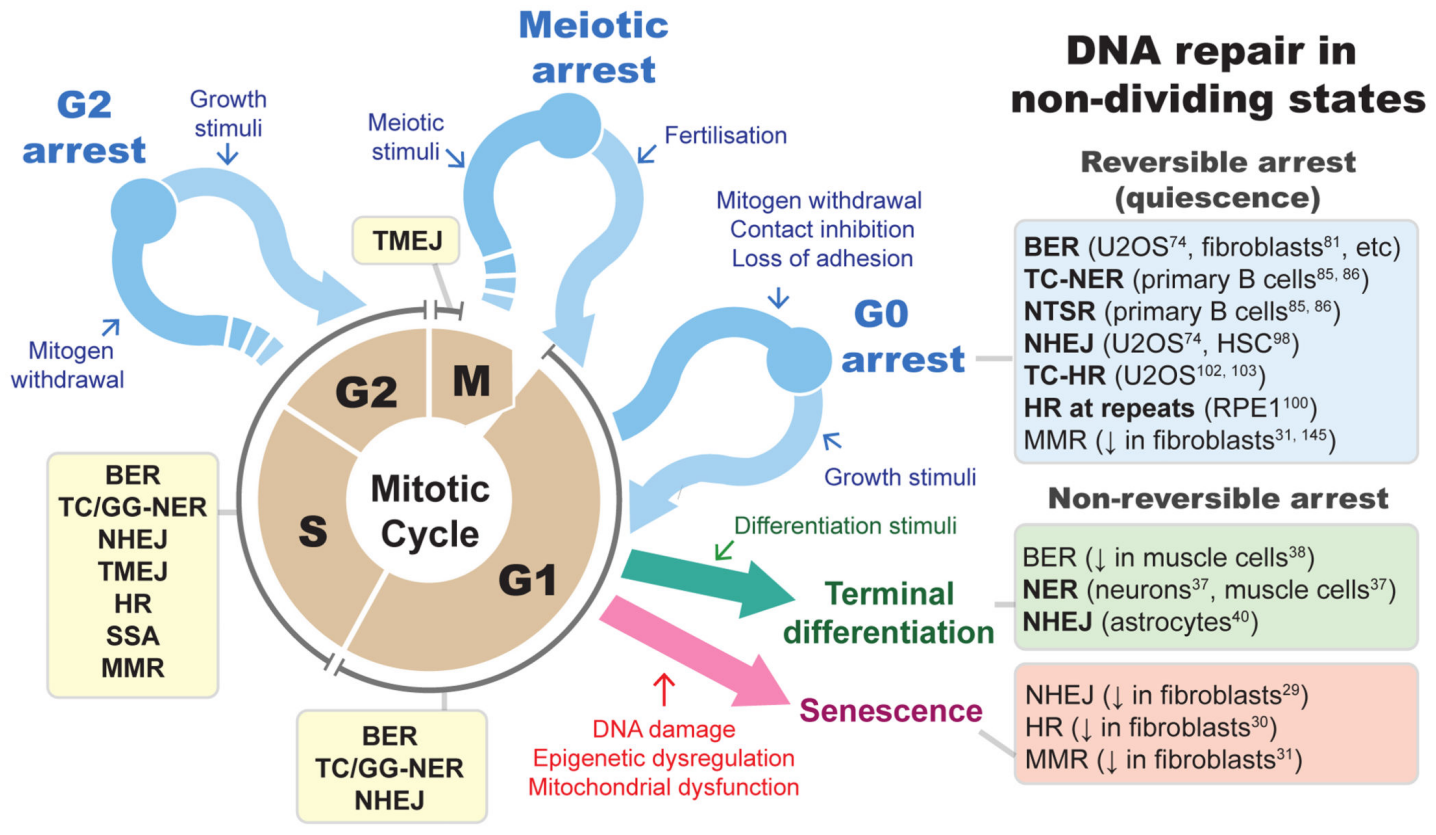
Overview of the repair mechanisms during the cell cycle and in different non-cycling states. The cell cycle and various non-cycling states are shown in conjunction with reported DNA repair mechanisms in each state (shaded boxes). There are three main non-cycling states, namely quiescence (commonly in G0 arrest, and less commonly in G2 and meiotic arrest; blue), terminal differentiation (green), and senescence (red). Unlike quiescence, the latter two states are widely regarded as non-reversible, i.e. cells cannot re-enter the cell cycle. Specific stimuli or lack of such stimuli can induce different non-cycling states. See Table 2 for more complete information about the reported DNA repair mechanisms in G0. NHEJ, non-homologous end-joining; TC/GG-NER, transcription coupled/global genome-nucleotide excision repair; NTSR, non-transcribed strand repair; BER, base excision repair; HR, homologous recombination; TC-HR, transcription coupled homologous recombination; MMR, mismatch repair; SSA, single-strand annealing; TMEJ, theta mediated-end joining.

Quiescence is a non-pathological state of cell cycle arrest in which cells retain the ability to re-enter the cell cycle without changing their original properties upon appropriate stimuli [1]. This state is distinct from other non-cycling states such as terminal differentiation and senescence (Fig. 1). Terminal differentiation, while also non-pathological, follows a series of signalling events, transcriptional reprogramming, and extensive structural changes that enable cells to perform their tissue-specific functions [2]. Exceptions include antigen-induced B- and T-cell differentiation, during which cells exit from quiescence and then enter a highly proliferative state for extensive diversification and amplification before they eventually stop dividing. Conversely, senescence is widely regarded as a pathological cellular state often resulting from accumulated DNA damage and genomic alternations including telomere shortening [3,4], albeit it has also been argued that entry into a senescent state can be seen as non-pathological in certain cell populations, for example during embryonic development [5].

Perhaps confusingly, all three non-cycling states exhibit active anti-proliferative signalling mechanisms, including the induction of cyclin-dependent kinases (CDKs) inhibitors (e.g. p16, p21, and p27), with other shared features such as lack of replicative DNA synthesis and a hypophosphorylated form of RB protein that restricts E2F-mediated transcription [1–3,6]. Such common molecular features likely make the precise definition and distinction of these cellular states difficult, and the terminology of non-cycling states is often used interchangeably. Additionally, it is debated that senescent cells can re-enter the cell cycle [7–9], although, by a strict definition, fully established senescent cells should remain in a non-cycling state under normal conditions [10]. In a similar vein, terminally differentiated cells may enter a cycling state only under pathological conditions (e.g. the loss of p53), or be induced to become pluripotent stem cells with an intact proliferative capacity upon forced transcriptional reprogramming (e.g. the induction of the Yamanaka factors OCT4, SOX2, KLF4, and c-MYC) [11].

Entering and maintaining quiescence, a state of reversible growth arrest as defined above, involves complex molecular processes that are dependent on tissue type and developmental stage [1,12]. Typically, quiescence is induced by the microenvironment, such as lack of access to growth factors and nutrients, and is maintained by transcriptional control to block cell cycle progression, for example *via* the DREAM (dimerization partner, RB-like, E2F and multi-vulval class B) complex that suppresses the expression of genes encoding cell cycle drivers, including cyclins, CDKs, aurora kinases, polo-like kinases (PLKs), DNA polymerases and E2F transcription factors [13,14]. Non-canonically, a more recent study proposes that the level of endogenous DNA damage also contributes to cell cycle exit to a quiescence state [15]. The transcriptional regulation in this mode of quiescence remains to be understood.

The proportion of quiescent cells in tissues diverges, as does the number of terminally differentiated and senescent cells [16]. As summarised in Table 1, stem cells often remain in a resting quiescent state for considerable amounts of time and their proportion varies across different tissues, being most abundant in testes, skeletal muscle, and adipose tissue [1,17]. However, quiescence is not restricted to stem cells, and many types of more differentiated cells also reside in a quiescent state. Examples of quiescent differentiated cells include fibroblasts, lymphocytes and oocytes [1]. They are also frequent in certain organs, such as the liver, where quiescent hepatocytes and quiescent hepatic stellate cells account for the majority of cells [18,19]. Most somatic cells enter quiescence from G1, also referred to as G0, but, under certain conditions, some cells are observed to be arrested in G2 or during the process of cell division. Indeed, several types of tissue cells and a fraction of pre-immune B (pre-B) lymphocytes are found in G2 [20,21], whereas all mammalian oocytes are arrested in meiotic prophase I [1, 22].

As many of the functions associated with cell division are absent in any of these quiescent states, they are largely spared from endogenous insults such as replication fork collapse, chromosome segregation errors, and telomere erosion. Additionally, active protection mechanisms are established in quiescent cells. These include chromatin reorganisation, involving changes in histone modifications and cohesin distribution, which is generally understood to induce chromatin compaction and transcriptional repression [23]. While their full effect on global chromatin architectures has yet to be characterised in human cells, studies in yeast suggest substantial topological reorganisation with increased intrachromosomal interactions, decreased inter-centromeric interactions, and increased inter-telomeric interactions [24]. Quiescent cells also often exhibit low metabolic activity, with substantial metabolic rewiring and increased clearance of mitochondria by mitophagy [25]. They rely less on oxidative phosphorylation and more on glycolysis, resulting in less ATP and fewer reactive oxygen species (ROS), thus reducing exposure to endogenous metabolic stress [13,25,26]. Despite these numerous changes, quiescent cells still suffer DNA damage arising from transcription required for basic cellular survival, residual endogenous metabolic stresses, and environmental stress, such as that induced by UV [27,28]. Different tissue types may be prone to different insults based on organ location and function, for example, UV damage is more likely to affect quiescent cells in the skin.

In this review, we outline the current understanding of DNA repair mechanisms during quiescence and discuss their implications for normal physiology and human diseases. With respect to those in other non-cycling states, there is a lack of clear evidence for active DNA repair in senescent cells, despite an apparent increase in DDR [29–34] (also reviewed in [3,35,36]), whereas some repair mechanisms have been reported in terminally differentiated cells [37–40]. This probably reflects the relative importance of DNA repair in cells in the respective states. We envisage that the need for accurate DNA repair is further enhanced in quiescent cells to retain their ability to re-enter the cell cycle with an intact genome and to produce healthy progeny cells. It is therefore highly relevant in diseases such as those associated with defective tissue regeneration and cancer, where disruptions in DNA repair mechanisms can contribute to the accumulation of mutations and genomic instability [41]. Of note, many reported findings on DNA repair mechanisms may be specific to tissue, cell type, model of quiescence duction, or type of insult, with potential biases due to the assays used (see Table 2). Hence, a comparison of their mechanisms across diverse tissue types may not be entirely appropriate. Nonetheless, understanding repair mechanisms in quiescent cells and comparing them with those in cycling cells should provide important insights into how they respond to injury and stress in the context of tissues.

## 2 Main endogenous sources of DNA damage during quiescence

### 2.1 Transcription-associated DNA damage

As a cell enters quiescence, the chromatin landscape changes drastically, with widespread histone deacetylation, chromatin compaction, and transcriptional repression [23,42,43]. However, far from being a global shutdown of transcription, quiescence represents a shift in focus for the cell and active transcription remains essential for viability, providing metabolic enzymes and ribosomal proteins [1]. While necessary, transcription is also inherently problematic as a potential source of endogenous DNA damage [44,45]. For example, transcription is associated with a higher incidence of oxidative damage at gene-regulatory elements and of deamination and secondary structure formation with a bias towards the displaced non-template strand [46]. Transcription also comes with torsional stress, for which specialised enzymes known as DNA topoisomerases help to relax the associated supercoiling by inducing strand breakage that must then be repaired [45,47,48]. Furthermore, analogous to the replication stress during DNA replication, the transcription machinery may encounter lesions that impede its progress and halt transcription elongation. Such lesions can be highly cytotoxic and must be removed promptly [44,45].

### 2.2 Oxidative stress

Rewiring metabolism during quiescence supports cellular respiration, anabolic and catabolic processes, which in turn produce ROS, such as superoxide radicals, hydrogen peroxide, singlet oxygen, and hydroxyl radicals. They are necessary for many cellular processes, including post-translational modifications, defence against pathogens, and cell-to-cell communication. In excess, however, this leads to a condition known as oxidative stress - a pathological process that affects most cellular structures, including nucleic acids [49]. Oxidative stress can therefore be highly mutagenic and pose a threat to overall genomic stability. The most well-studied lesion associated with oxidative damage is the formation of 8-oxo-7,8-dihydroguanine (8-oxoG). If left unrepaired, such modifications can disrupt transcription factor binding, alter transcription patterns, and affect secondary structures including G-quadruplexes, and consequently affect cell viability [50–52].

## 3 Cellular models for studying DNA repair during quiescence

Experimental study of quiescence often involves manipulating cell culture conditions to mimic the microenvironment that prompts cells to enter a non-dividing state, such as serum deprivation, restricted access to growth factors and certain nutrients, contact inhibition, loss of adhesion, or hormone withdrawal for hormone-dependent cells [1, 53–57]. Other ways of inducing quiescence include exposure to cell cycle inhibitors or DNA-damaging agents, although these conditions are somewhat invasive and often induce senescence or cell death. A quiescence state is commonly monitored by the expression of CDK inhibitors (p21, p27), hypophosphorylated RB, and primary cilia, while Ki-67 protein levels and DNA synthesis, defined by the level of proliferating cell nuclear antigen (PCNA) and nucleotide analogues incorporation, have been used as negative markers [1,16,58–62]. The relative importance, sensitivity, and specificity of each marker are context-dependent. As discussed above, these markers are also shared by cells undergoing senescence and terminal differentiation and, hence caution should be exercised in using them alone to define quiescence. This issue can be addressed by simultaneously evaluating the absence of markers of senescence or terminal differentiation. Quiescence can alternatively be viewed as a dynamic state, in which the proteomic and transcriptomic landscape can vary between cells and over time. In this context, quiescence cannot be clearly defined but instead exists as a spectrum between cycling and senescence, where a cell can exist at different depths of quiescence, and where the threshold for re-entry into the cell cycle increases the deeper the state of quiescence is [6,63].

Despite these challenges in defining quiescence in physiological conditions, efforts have been made to study DNA repair during experimentally induced quiescence. The most commonly used cell types in the field include human fibroblasts, epithelial cells, and various stem cells including haematopoietic stem cells (HSCs) and neural stem cells. To study DNA damage response (DDR) during quiescence, cells or model organisms are typically exposed to exogenous DNA damage such as UV light, ionizing radiation, inducible nuclease systems, or chemotherapeutic drugs. There is a wide range of assays to study DNA repair [64, 65], including immunofluorescent detection of DNA repair proteins (e. g., RAD51, 53BP1) or specific post-translational modifications (e.g., phospho-histone H2A.X) as molecular markers of repair processes. Other readouts include western blotting for DNA damage checkpoint proteins, measurements of repair products using reporter systems [66–69], fluorescence in situ hybridization (FISH) [64], and sequencing approaches [70,71].

## 4 Regulation of repair pathways during quiescence

While quiescence-induced transcriptional programmes typically limit the induction of DNA damage and prevent premature cell death [1], they also downregulate the expression of many DNA repair factors, thereby favouring certain repair mechanisms in quiescent cells [72–74]. Among these, activities of base excision repair (BER), nucleotide excision repair (NER), and double-strand break (DSB) repair pathways have been reported. In the following section, we outline these pathways and highlight some quiescence-specific regulatory mechanisms, focusing on mammalian cells.

### 4.1 Regulation of BER pathway

BER is responsible for the removal of non-helix-distorting small base lesions, such as those caused by spontaneous decay of DNA (e.g., deamination from spontaneous hydrolysis), enzymatic action (e.g., cellular deaminases), oxidative damage from metabolic processes, or alkylation reactions (e.g., methylation) [75]. BER is initiated by a number of specialised DNA glycosylases capable of both recognising different lesions and removing the affected bases [75,76]. This leaves an abasic site, which can then be processed to form a single-strand break or a single-nucleotide gap. Dedicated DNA polymerases subsequently fill the gap, which is then followed by sealing of the resulting nick. Interestingly, the resolution of long-patch BER, which complements canonical short-patch BER, involves an essential DNA replication protein, PCNA, which aids in recruiting DNA polymerases and flap excision factors [77]. Indeed, PCNA is shown to be actively recruited to DNA upon DNA damage even in non-cycling cells such as quiescent fibroblasts [78,79].

As mentioned above, specific DNA glycosylases are required for the recognition of different lesions in both short- and long-patch BER. For example, the N-methylpurine DNA glycosylase (MPG) primarily recognises alkylated bases whereas the Nei-like (NEIL) family of DNA glycosylases has a preference for oxidised bases [75,76,80]. While studies comparing the levels of different glycosylases are limited, there is evidence for some differential regulation between quiescent cells and non-quiescent cells. For example, in human fibroblasts, NEIL1 expression is higher in quiescence cells than in cycling cells, whereas NEIL3 expression exhibits the opposite trend [81]. Although it is not completely clear why there is a difference in the expression of the two glycosylases in quiescence, it was previously shown that NEIL1 acts preferentially on lesions located on double-stranded DNA, whereas NEIL3 prefers lesions on single-stranded DNA arising from DNA replication [80]. It may be hypothesised that the types of lesions which NEIL1 recognises, but NEIL3 does not, are more frequent in quiescent cells.

### 4.2 Regulation of NER pathway

**Fig. 2:**
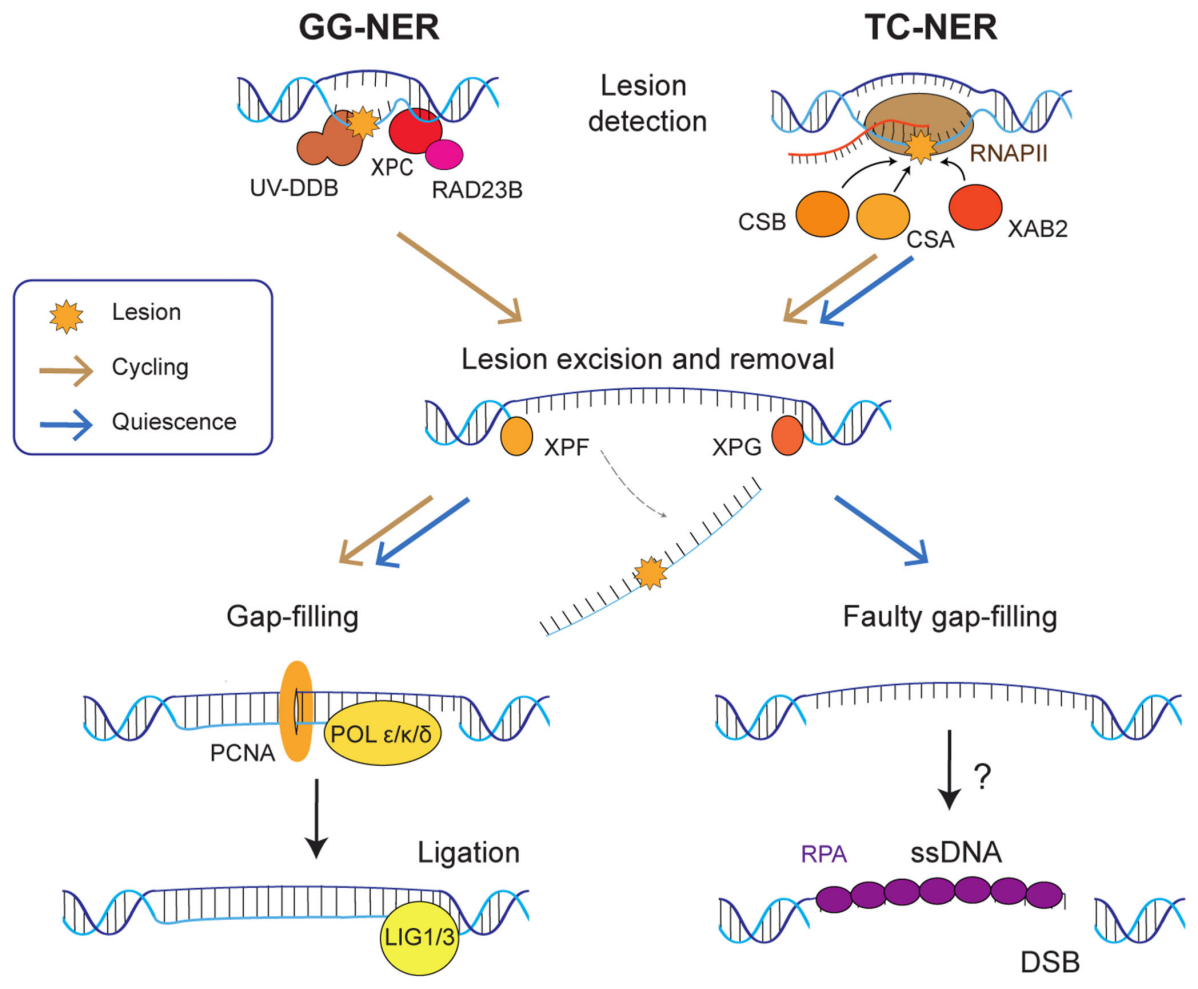
NER mechanisms in cycling and quiescent cells. Global genome (GG)- and transcription coupled (TC)-NER differ in lesion recognition, but lesion excision and removal are carried out by the same set of proteins, crucially by structure-specific endonucleases XPF and XPG. Generated ssDNA gaps are filled and sealed in a manner dependent on replicative factors, including DNA polymerases, PCNA, and ligases. Quiescent cells are inefficient in gap-filling and nick-sealing, leading to either the formation of ssDNA or DSBs, triggering DDR. Brown arrow indicates presence of a functional pathway in cycling cells. Blue arrow indicates presence of a functional pathway in quiescent cells.

NER is the primary pathway used to repair helix-distorting bulky lesions including UV-induced photoproducts such as pyrimidine dimers, oxidative lesions, and various adducts [82]. NER can be divided into two distinct repair pathways, namely global genome NER (GG-NER) and transcription-coupled NER (TC-NER) [82] (Fig. 2). GG-NER is known to occur anywhere in the genome where lesions are directly detected by Xeroderma Pigmentosum Group C (XPC) and UV-damaged DNA binding protein (UV-DDB). In contrast, TC-NER is restricted to transcriptionally active regions as it relies on the detection of lesions on RNA polymerase II (RNAPII) and accessory factors such as Cockayne syndrome proteins (CSA, CSB) and XPA-binding protein 2 (XAB2). In both pathways, the lesion and section of the strand surrounding the lesion are excised before the resulting gap is filled by replicative DNA polymerases (Pol ε/δ) or a translesion polymerase (Pol κ)-mediated repair synthesis aided by PCNA and the nick is sealed by DNA ligases [82].

Similar to most other DNA repair pathways, the expression of NER factors is downregulated during quiescence, in part mediated by the DREAM complex [74]. Another factor contributing to the reduced efficiency of NER during quiescence has been linked to the decreased phosphorylation of ubiquitin-activating enzyme 1 (UBE1), although how UBE1 regulates NER is not entirely clear [83]. Interestingly, in quiescent fibroblasts, the efficiency of NER was also found to be affected by the circadian clock, which alters the chromatin state through histone acetylation [84]. Despite the lower efficiency of NER in quiescent cells, studies show that NER is still active and is essential for the survival of quiescent cells in response to UV-induced damage [85]. Notably, in quiescent pre-B lymphocytes and terminally differentiated macrophages, TC-NER but not canonical GG-NER was found to remain active [83,86]. Regardless, the non-transcribed strand of actively transcribed regions was still found to be efficiently repaired in both mouse and human cells, and this repair was deemed non-transcribed strand repair (NTSR) or transcription-domain/differentiation-associated repair (DAR) [83,86,87]. Although the molecular mechanisms of NTSR and its activity in cycling cells remain largely unclear, Hyka-Nouspikel et al. identified that GG-NER factor XPC is required for this repair process.

This channelling of NER to repair transcriptionally active genes might be a necessary regulation for the cell to efficiently utilises the remaining repair factors available to maintain the integrity of these regions, allowing the cell to survive in quiescence when exposed to stress. It might also simply be a secondary adaptation to transcription being a major source of DNA damage. Whether TC-NER or NTSR also plays a role in repairing actively transcribed but non-coding regions during quiescence, such as the centromere [88], is not known. In contrast to transcribed regions, lesions may then accumulate in genes that are not actively transcribed during quiescence and become a source of replication stress and mutagenesis when the cell re-enters the cell cycle [86]. Further studies are needed to clarify whether GG-NER is suppressed in favour of TC-NER in other cell types and whether damage accumulated in non-transcribed regions is only repaired upon re-entry into the cell cycle.

### 4.3 Regulation of DNA double-strand break repair pathways

DNA strand breaks can be induced in a number of ways, including during the processes of transcription and DNA repair. Indeed, NER intermediates, if unprocessed promptly, can result in long-lasting single-stranded DNA breaks (SSBs) and more deleterious DSBs [89,90] (Fig. 2).

**Fig. 3:**
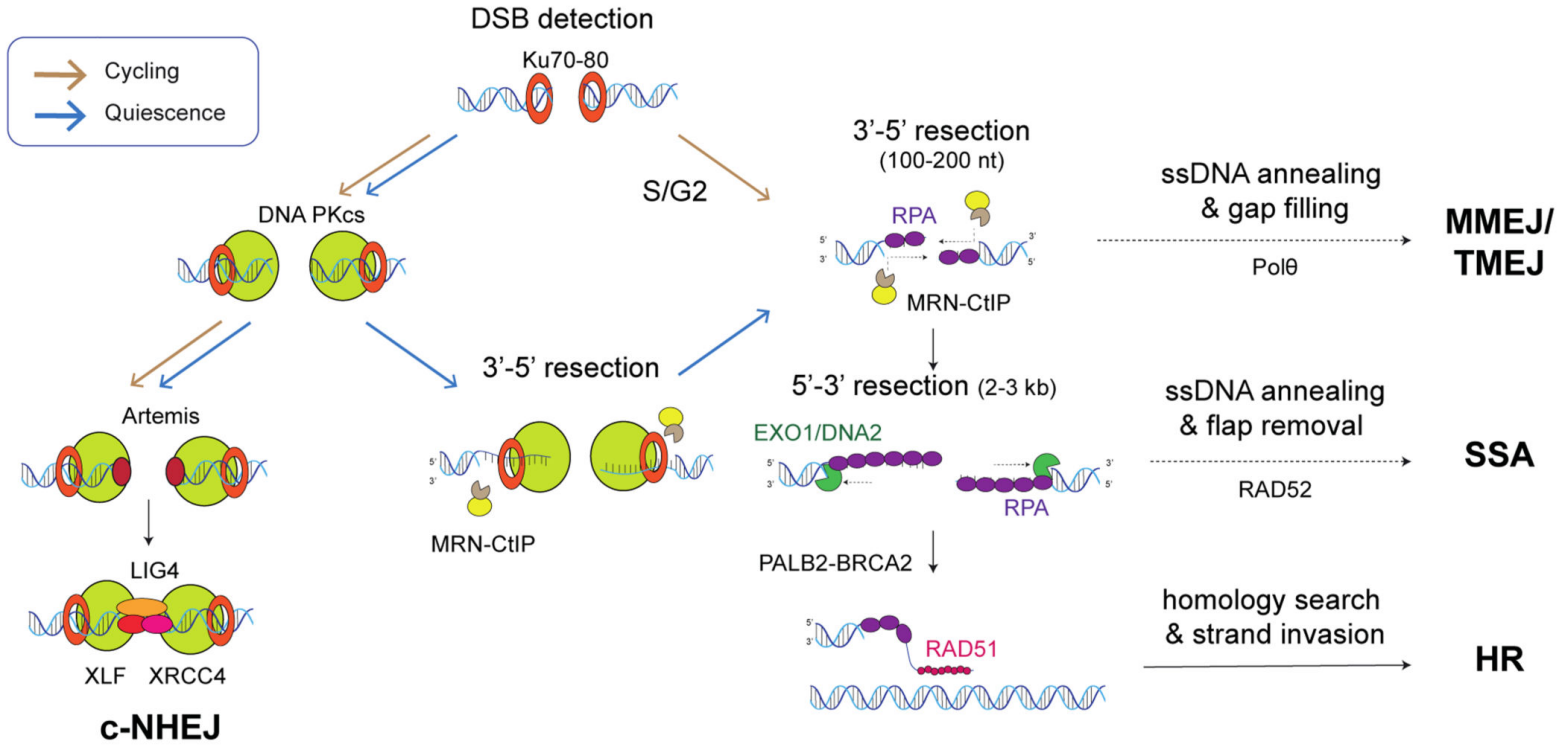
DSB repair pathways in cycling and quiescent cells. DSBs are largely repaired by non-homologous end-joining (NHEJ), homologous recombination (HR), or alternative pathways, i.e. microhomology-mediated end-joining/theta-mediated end-joining (MMEJ/TMEJ) or single-strand annealing (SSA) in a cell cycle- and resection-dependent manner. While DNA-PKcs catalyses NHEJ in cycling cells, quiescent cells utilise this enzyme to promote MRN-CtIP-dependent resection, potentially leading to resection-dependent repair. Brown and blue arrows indicate presence of functional pathways in cycling and quiescent cells, respectively.

The repair of DSBs is mainly mediated by two repair pathways, non-homologous end-joining (NHEJ) and homologous recombination (HR) [91] (Fig. 3). The former is the predominant mechanism in most human cells, accounting for around 80 % of DSB repair events in asynchronous cells [92]. In NHEJ, the DSB is first recognised by the Ku70-Ku80 heterodimer. This serves not only to protect the DSB from nucleolytic activity but also to recruit downstream NHEJ machinery including DNA-PKcs and Ligase IV (LIG4), which in turn fuses two ends together [93,94]. In contrast, during HR, the MRE11 nuclease component of the MRN complex initiates resection at the 5’ ends of DSBs, a process activated by CtBP-interacting protein (CtIP) [91]. This generates 3’ single-strand DNA overhangs, which are bound and protected by replication protein A (RPA). Typically, the BRCA2-PALB2 complex then displaces RPA and assists the loading of the essential recombinase RAD51, facilitating RAD51 filament formation, homology search, and strand exchange - identifying a complementary sequence to use as a template for DNA repair synthesis.

In cycling cells, HR is generally considered to be restricted to the S/G2 phase of the cell cycle due to the requirement of a homologous sequence, usually the sister chromatid, as a repair template. Key regulators of this process include CDKs, which phosphorylate CtIP to initiate resection. While HR is slow and elaborate, it can offer error-free repair of DSB. This is in contrast to NHEJ, which is active in both G1 and S/G2 as it does not require a template for repair, but it is thus considered to be more error-prone. Other pathways for DSB repair include single-strand annealing (SSA) and alternative end-joining/microhomology-mediated end-joining (alt-EJ/MMEJ), also known as theta-mediated end-joining (TMEJ), which require two single-strand overhangs with homology, promoted by RAD52 and a mutagenic DNA polymerase Pol θ respectively. These pathways are considered to be the most unfaithful repair choices and act as backup repair pathways when HR or NHEJ are compromised, and their activity is dependent on the cell cycle phase, chromatin context, and extent of resection. An example of this is the preferred use of MMEJ/TMEJ in mitotic cells [95].

As with many other types of DNA repair, overall DSB repair capabilities are commonly seen to be downregulated during quiescence. For example, the efficiency of DSB repair in non-tumorigenic mammary epithelial MCF10A cells was found to be less efficient during G0, leading to persistent accumulation of DDR markers, γ-H2A.X, and 53BP1 [96]. On the other hand, studies using HSCs have demonstrated that NHEJ is the dominant pathway choice for DSB repair despite the potential mutagenic consequences of this choice [97,98]. This can be explained by the fact that, in addition to the lack of a suitable HR repair template, namely a replicated sister-chromatid, certain HR factors are down-regulated in pre-B cells by the DREAM complex, limiting DNA resection and HR efficiency [73]. However, the lack of a faithful DSB repair mechanism could be seen as dangerous given the need for quiescent cells to maintain genomic integrity for cell cycle re-entry, since cells must be able to re-enter the cell cycle and proliferate with an intact genome.

**Fig. 4:**
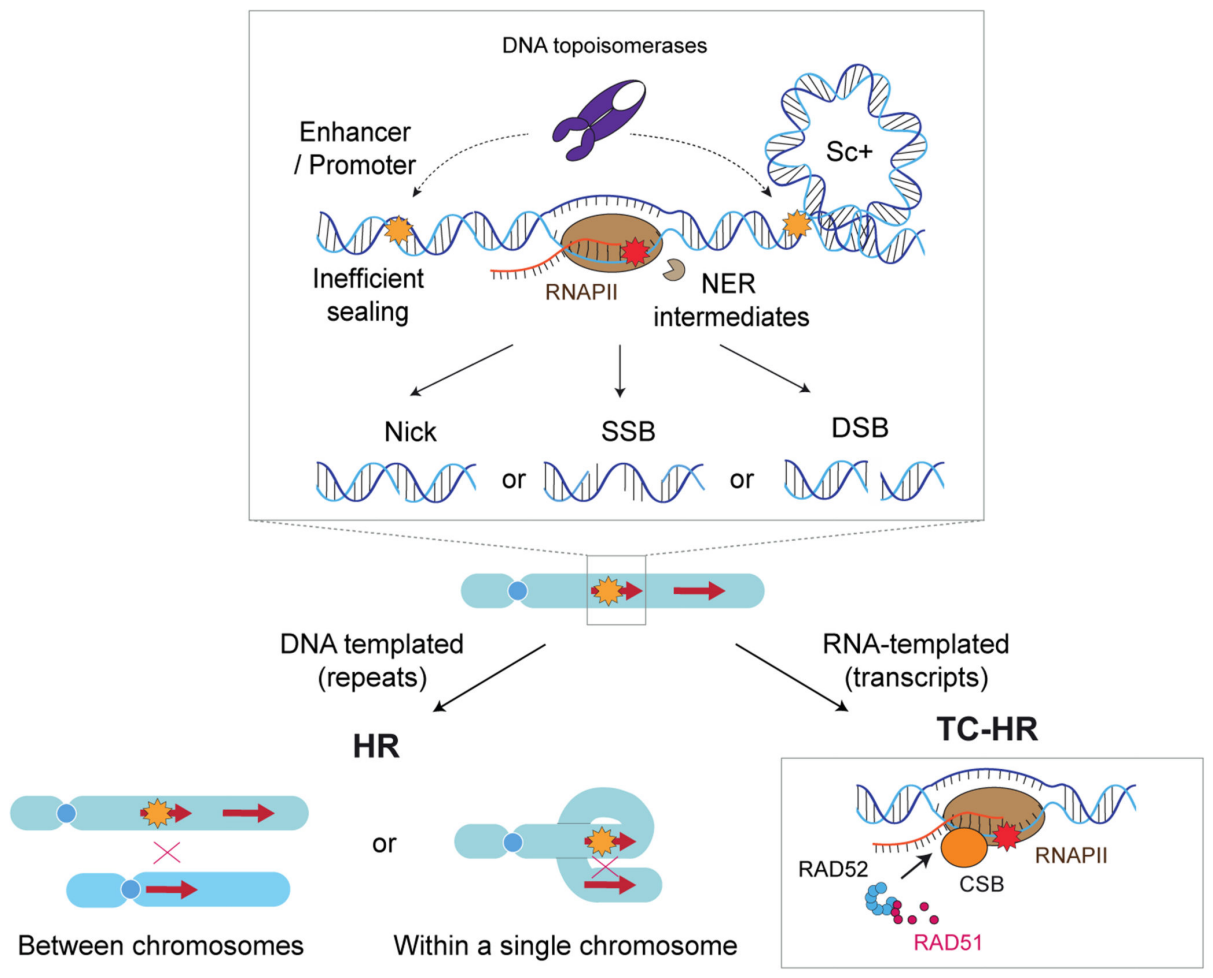
Potential triggers and mechanisms of HR in quiescent cells. Nicks, SSBs, and DSBs are all known potential substrates for repair by HR. Potential sources of such substrates in quiescent cells include intermediates of NER due to the inefficient gap-filling and nick-sealing processes. Alternatively, a type II topoisomerase, TOP2B, which is active during quiescence, induces transient DNA breaks, which is important to regulate transcription or resolve associated positive supercoils (Sc+). HR repair can then be carried out between chromosomes or within a single chromosome if in a repetitive region of the genome, or TC-HR if in an actively transcribed region.

Intriguingly, there is emerging evidence that HR may remain active in certain conditions and in certain genomic locations. For example, in HaCaT keratinocytes, the key HR enzyme RAD51 was shown to be important in protecting not only cycling but also quiescent cells from genotoxic stress including UVB-radiation and transcriptional stress independently of NER [99]. Further, our recent work has found that RAD51 suppresses the accumulation of intrinsic DNA breaks at centromeres in quiescent non-cancerous retinal pigment epithelial (RPE1) cells and that this function of RAD51 is dependent on its strand-exchange capabilities [100]. Thus, although NHEJ is dominant in repairing DSBs in quiescent HSCs, it is becoming increasingly clear that HR plays a role in repairing DNA and cell survival in quiescent epithelial cells. It can be postulated that, even in the absence of a sister chromatid, recombination may occur in repetitive regions of the genome, e.g. at centromeres and telomeres, or using other repeats intra- or interchromosomally as a template for repair (Fig. 4). One of the first lines of evidence for such a process came from Ghandi et al., demonstrating that homologous chromosomes, required for HR, make contact at DSB sites in quiescent cells [101].

While cells normally enter G0 by exiting the cell cycle before the G1 restriction checkpoint, recent studies have highlighted that there are exceptions. For example, within a population of MCF10A cells, some cells appear to exit the S/G2 phase and enter a quiescent state. Such cells would have finished replicating their DNA and therefore would be able to carry out HR repair using an available sister chromatid as a repair template [22]. However, it is currently unknown whether the canonical HR mechanism, which utilises homologous DNA for repair, remains active in such cells. Alternatively, HR repair may occur in transcriptionally active regions by employing RNA as a template [102,103]. This process, referred to as transcription-coupled HR (TC-HR), is dependent on transcription at the site of a DNA break and involves stalling of RNAPII, R-loop formation, and detection of such R-loops by CSB. This in turn recruits RAD52 which, together with CSB, loads RAD51 in a BRCA2-independent manner (Fig. 4). This latter pathway is a relatively recently discovered form of HR shown to be active in quiescent cells and seems to be another mechanism by which quiescent cells can protect and maintain actively transcribed regions, similar to TC-NER and NTSR.

As mentioned above, in the context of DSB repair, DNA resection is a key determinant of whether cells use NHEJ or homology-mediated repair pathways such as HR, MMEJ/TMEJ, or SSA. However, there have been conflicting reports on the extent to which DNA resection occurs in quiescent cells. Li *et al*. demonstrated that in immortalised fibroblasts, CtIP is downregulated during quiescence, preventing resection [104]. Consistent with this, Chen *et al*. also observed that CtIP-dependent resection is suppressed by the DREAM complex in quiescent pre-B cells [73]. In contrast, a more recent study using MCF10A showed that DNA-PKcs can uniquely promote CtIP- and MRE11-dependent resection upon ionising radiation during quiescence [105]. This is surprising given the well-described role of DNA-PKcs in cycling cells, where it prevents rather than promotes resection [93]. It remains unclear why this is the case and raises the possibility that other DDR factors may also take on new roles during quiescence. The conflicting evidence between these two studies on CtIP-dependent resection in quiescence may also be explained by differences in the cell types studied.

## 5 Implications for human disease and therapeutic approaches

Failure to repair DNA breaks during quiescence can lead to reduced stem cell populations and inadequate tissue repair and regeneration [12]. Even if these cells could return to a cycling state upon appropriate stimuli, they are likely to leave their progeny cells with genomic alterations and instability. While quiescent cells mitigate this risk by reducing the overall metabolic rate and by chromatin reorganisation, DNA repair capability is often attenuated, and the lifespan of quiescent cells is generally long. Indeed, it has been shown that quiescent HSCs can be compromised by an accumulation of DNA damage, highlighting the importance of retaining adequate DNA repair capacity [72]. This can eventually lead to impaired production of functional progeny and possibly contribute to the emergence of malignant clonal populations [72,98,106–108] or organ failure (incl. bone marrow failure) from the depletion of cells with replicative potential [108–110]. Mutations in DNA repair genes are also tightly linked with increased levels of DNA damage in other cell types, including quiescent oocytes, ultimately leading to diminished oocyte reserves, infertility, and premature menopause [111–113]. For most tissue types, it is not well characterised what the impact of DNA repair defects is on the quiescent cell populations, but it is well known that even organs like the brain with predominantly terminally differentiated cells have some degree of cell turnover. Defects in DNA repair might therefore affect the population of quiescent neuronal stem cells, for example in the hippocampus, one-third of which is replaced in adulthood [114].

Beyond the normal physiology of tissue and organs in human bodies, it is beginning to be recognised that a certain population of cancer cells goes into a non-cycling state, referred to as cancer dormancy [115,116]. These dormant cancer cells can exist in the primary tumour and in distant secondary sites and persist for years before returning to a cycling state and driving relapse. This is explained by the ability of cancer cells to enter quiescence and evade, for example, immune detection [117] and chemotherapy targeting actively dividing cells [115,116]. Indeed, DNA damage-inducing agents have been widely used as chemotherapeutic agents in the treatment of many human diseases, including cancer. Their use can be understood by viewing cancer as an abnormal state of uncontrolled proliferation with an attenuated response to growth inhibitory cues (e.g., due to defects in the RB- or p53-pathways) [41], such that cancer cells with excessive DNA damage are eliminated during defective DNA replication or cell division. However, it has become clear that this is an oversimplified picture that neglects the heterogeneity of many tumours. Even in cases where the initial response is good, resistance and relapse can develop. Furthermore, many tumours, including slow-growing melanomas and glioblastomas, have low response rates to conventional chemotherapy [118–120]. Here, several lines of evidence suggest that quiescent subpopulations correlate with poor response to therapy, recurrence, and overall survival rate [121–123]. However it has also been shown that some quiescent cells are more sensitive to radiotherapy than cycling cells, and some of the observed differences are likely to reflect DNA repair capabilities as well as changes to the apoptotic threshold [1,124,125], although the evidence supporting the assumption that dormant cancer stem cells have the same status as normal stem cells remains a matter of debate [115].

To address the issue of cancer cell dormancy, different strategies have been proposed (reviewed in [116]). If dormant cancer cells can be kept in a long-term state of quiescence, then perhaps it would be possible to prevent disease relapse. One example of an experimental approach taken includes treatment with a dormancy-inducing factor like TGF-β [126]. The opposite approach can also be taken, where rather than keeping cells in G0, these would be forced to re-enter the cell cycle, and thus resensitised to genotoxic chemotherapy as well as potentially immunotherapy and radiation. As cycling cells generally have higher repair capacity, this option might be most suited to cancer cells with mutations in specific DNA repair pathways. Forcing non-cycling cancer cells to start dividing also carries a risk of exponential tumour growth and poses a dilemma should such cells also have acquired other ways of desensitising themselves. A third option, perhaps the most attractive option in theory but also the most challenging in practice, would be specific targeting and eradication of dormant cancer cell populations. This can be achieved by a simultaneous inhibition of survival pathways maintaining dormancy and pathways enabling escape from dormancy [127,128]. From a DNA repair standpoint, the increased reliance on specific repair pathways might also present opportunities to selectively target quiescent cancer cells. However, minimising the effects on healthy cells is key, and strategies will need to take into account the mutational status and DNA repair capabilities of dormant cancer cells to find specific weaknesses. Altogether, these three approaches have been termed the “Sleeping strategy,” “Awakening strategy,” and “Killing strategy” – to maintain, awaken, or eradicate dormant cancer cells (including disease-maintaining cancer stem cells) respectively [127]. Indeed, analogous to the issue of quiescent cancer cells, drugs aimed at clearing tissues of senescent cells are being studied in the context of preventing or treating associated with an excess of senescent cells, e.g., in Alzheimer’s disease and chronic kidney disease [129]

## 6 Conclusions and future directions

Much remains unknown about the regulation of DNA repair in cells in a quiescent state that is not a single homogeneous phase. The transcriptional programmes of quiescent cells may differ depending on multiple factors, including the signal used to induce quiescence, the length of time the cell remains in quiescence, and the type of cell that is in quiescence, plausibly with intrinsic preference for certain repair pathways. Indeed, the diverse epigenotypes and transcriptional landscapes likely affect the phenotypes seen, and the molecular events mapped. It is yet to be understood how the regulation of DNA repair may be affected by these different quiescent states. Additionally, most studies have examined the accumulation of mutations and DNA damage using exogenous sources of breaks, such as UV and ionising radiation. As such, the observed phenotype might be skewed due to the exaggerated levels of DNA damage that exceed the cells’ repair capabilities in a way that is unlikely to occur *in vivo*, where even an attenuated DDR can meet the physiological needs. There is a pressing need to comprehend whether the cell also responds in a similar fashion and uses the same DNA repair pathways to repair breaks from endogenous sources of breaks.

The current understanding is that DNA repair factors are down-regulated in quiescent cells, presumably because there is less need for these factors due to fewer sources of breaks compared to cycling cells. It may seem paradoxical that cells downregulate DNA repair pathways when faced with the challenge of maintaining genomic integrity over extended periods of quiescence. However, it has become evident that this might be an oversimplification and that many repair factors are not completely absent, but instead that levels are proportionally decreased as the need has decreased, and some factors may even have different roles in quiescence than their canonical roles in DNA repair in cycling cells. For example, several factors involved in GG-NER in cycling cells are redirected to NTSR, and DNA-PKcs, which control NHEJ in cycling cells, can promote resection, and potentially HR. It would also be important to note that homology-mediated repair could be initiated without resection. HR can be triggered by SSBs, nicks, and gaps, where single-stranded DNA required for strand invasion can be generated by helicase unwinding of DNA [130,131], whereas MMEJ or SSB could occur on extended single-stranded DNA formed independently of resection. These DNA substrates may arise as repair intermediates during NER, suggesting potential crosstalk between these repair pathways, as in cycling cells where the NER factor XPG has been shown to promote HR [132]. It is possible that other DNA repair factors may also have different functions in quiescence, and it will be important to systematically analyse these repair factors in detail using a wide range of assays in quiescent cells. It is also noteworthy that some cells have also been shown to re-enter the cell cycle to repair DNA damage by HR [15,72] and intermittent exits from quiescence to generate transit-amplifying cells may be a way around the increased reliance on error-prone repair often perceived in quiescence.

Understanding the cell biology of quiescence and the DNA repair mechanism in these cells should also provide important insights into human diseases, such as difficult-to-treat dormant cancers, which avoid the toxic effects of many chemotherapeutic agents that rely on the cell cycle for their action and use dormancy as a route to evade the immune system. With the knowledge of DNA repair in normal quiescent cells and dormant cancer cells, existing chemotherapies could be repurposed or used in combination therapy with drugs that target specific repair pathways to prevent dormant cancers from returning.

## Figures and Tables

**Table 1:** Examples of quiescent cells and their locations in the body.

Cell type	Quiescence (%)	Organ/Tissue	Terminally Differentiated (lifespan)	Reference
Oocytes	100 % (?)	Ovary	Oocytes (50 years)	
Spermatogonial stem cells	>95 %	Testis	Sperm (2.5 months)	[133]
Neural stem cells	~88 % (mice)	Brain	Neuron (lifelong?)	[134]
Epidermal stem cells	~90 %	Skin	Keratinocyte (2 months)	[135]
Skeletal muscle cells	>99 % (mice)	Muscle tissue	Skeletal muscle cells (15 years)	[136]
Preadipocytes	~94.5 %	Adipose tissue	Adipocytes (8 years)	[137, 138]
Fibroblasts	N/A	Connective tissue	Postmitotic fibroblasts (2 months)	[139, 140]
Lymphocyte	~70 %	Lymph nodes, Spleen, etc	B/T lymphocytes, Natural killer cells (weeks to years).	[141, 142]
Haematopoietic stem cells	~70 %	Bone marrow	Erythrocyte (120 days), Neutrophil (5 days), Macrophage (several months), B/T lymphocyte etc	[143, 144]
Hepatocyte	>99 %	Liver	Hepatocyte (400 days)	[18]

**Table 2:** Reported DNA repair pathways and factors during quiescence.

Repair pathway	Sub repair pathway	Cell type	Relative activity in quiescence	Differentially regulated factors	References
BER	Short patch-BER	Embryonic fibroblast, Osteosarcoma, Keratinocytes, Breast epithelial cancer cells, Primary fetal lung fibroblasts	?	hNEIL1↑ hNEIL3↓ FEN1↓	[73,74,80,81]
	Long patch-BER	N/A	?	N/A	
NER	GG-NER	Primary B lymphocytes	Low	N/A	[85,86]
	TC-NER	Primary B lymphocytes	High	N/A	[85,86]
	NTSR	Primary B lymphocytes	High	N/A	[85,86]
MMR	-	Colonic fibroblasts, Embryonic lung fibroblast	Low	MSH2 ↓MLH1↓	[31,145]
NHEJ	-	Hematopoietic stem cells, Osteosarcoma cells	High	XRCC4 ↓	[73,74,90,98]
HR	Canonical HR	Osteosarcoma cells, Breast epithelial cells, Pre-B lymphocytes (Mouse)	Low	BRCA1 ↓ BARD1 ↓ BLM↓ RAD51 ↓ FANCD2 ↓ CtIP↓ PALB2↓	[72–74]
	TC-HR	Osteosarcoma cells	High	?	[73,74,102, 103]
SSA	**-**	Fibroblasts, Osteosarcoma cells	Low	CtIP↓	[73,74,103, 104]
TMEJ/MMEJ	**-**	Fibroblasts, Osteosarcoma cells	Low	CtIP↓	[73,74,103, 104]

* Denote humans / cell lines unless specified.

## Data Availability

No data was used for the research described in the article.
